# NLRX1 accelerates cisplatin-induced ototoxity in HEI-OC1 cells via promoting generation of ROS and activation of JNK signaling pathway

**DOI:** 10.1038/srep44311

**Published:** 2017-03-13

**Authors:** Haiyan Yin, Gaoying Sun, Qianqian Yang, Chen Chen, Qi Qi, Haibo Wang, Jianfeng Li

**Affiliations:** 1 Department of Otolaryngology-Head and Neck Surgery, Shandong Provincial Hospital Affiliated to Shandong University, Jinan, 250021, P.R. China; 2 Department of Pathology and Pathophysiology, Shandong University, Cheeloo Healthy Science Center, Jinan, 250012, P.R. China; 3 Shandong Provincial Key Laboratory of Otology, Jinan, 250021, P.R. China

## Abstract

Nucleotide-binding domain and leucine-rich-repeat-containing family member X1 (NLRX1), located in mitochondria, can recognize cytoplasmic pattern recognition receptors and is tightly related to reactive oxygen species (ROS) production, mitochondrial function, apoptosis and inflammation. The present study was designed to explore whether NLRX1 expresses in HEI-OC1 cells and, if so, to investigate the possible correlations between NLRX1 and cisplatin-induced ototoxity *in vitro*. Here, we report that NLRX1 was specifically localized to mitochondria in the cytoplasm of HEI-OC1 cells and its expression was increased concurrent with the increase of ROS production and occurrence of apoptosis in HEI-OC1 cells in response to cisplatin stimulus. NLRX1 overexpression led to a higher apoptosis in HEI-OC1 cells treated with cisplatin, whereas, NLRX silencing decreased cisplatin induced apoptosis. Mechanistic studies showed that NLRX1 activated mitochondrial apoptosis pathway as well as promoted ROS generation and JNK activation. Either inhibition of ROS generation or JNK signaling significantly prevented NLRX1-mediated mitochondrial apoptosis in HEI-OC1cells. In addition, NLRX1 expression was confirmed in cochlear explants. The findings from this work reveal that NLRX1 sensitizes HEI-OC1 cells to cisplatin-induced apoptosis via activation of ROS/JNK signaling pathway, suggesting that NLRX1 acts as an important regulator of the cisplatin-elicited ototoxity.

Cisplatin, the chemotherapeutic agent widely used in the treatment of a broad spectrum of tumors^1^, is limited in its clinical application due to its severe side effects, including the ototoxicity and nephrotoxicity^2^. Ototoxicity is one of the most severe adverse effects of cisplatin administration manifesting irreversible, accumulative, and bilateral hearing impairment^3^,^4^,^5^,^6^. However, the mechanisms responsible for cisplatin-induced ototoxicity are not yet fully understood and little is known about possible candidates that may be involved in this ototoxic process.

In recent years, the role of NLRX1, particularly its pivot contribution to immune, inflammation and cancer, has generated considerable interest. NLRX1 is a member of the evolutionarily conserved Nucleotide-binding and oligomerization domain (NOD)-like receptors (NLRs) family. NLRs are intracellular pattern recognition receptors (PRRs) that recognize pathogen-associated molecular patterns (PAMPs) and danger-associated molecular patterns (DAMPs) that function in innate immunity^7^,^8^,^9^,^10^. NLRX1 is the first NLR protein shown to be localized at the mitochondria^11^. The majority of researches have been directed towards the role of NLRX1 in regulating innate immunity and inflammation^9^,^12^. Except for mediating inflammation, recent studies have suggested that NLRX1 could regulate virus-induced autophagy and ROS production, mitochondrial function, cell death, and carcinogenesis^12^,^13^,^14^,^15^. Specifically, NLRX1 interacts with a mitochondrial matrix protein UQCRC2, a component of the respiratory chain complex III, suggesting that NLRX1 influence mitochondria ROS production^11^,^16^. This hypothesis was further supported by Soares *et al*. using NLRX1-knockout murine embryonic fibroblasts (MEFs), which displayed blunted tonic levels of ROS in resting condition compared to wild type MEFs^14^. Also, Tattoli *et al*. demonstrated that NLRX1 overexpression enhanced the formation of mitochondrial ROS production to activate NF-kappaB and JNK pathways^17^. Moreover, recent findings from several groups have identified a role of NLRX1 in the control of cell death in different cell types in response to different stimulus, such as regulating mitochondrial dynamics and neuronal death, accelerating apoptosis in different cell types^10^,^15^,^18^,^19^.

Mounting evidence has accumulated to demonstrate that cisplatin-induced ototoxicity is closely related to the ROS accumulation, mitochondrial dysfunction, apoptosis and inflammation-induced damage to the cochlea cells^20^,^21^. It has been documented that a moderate increase in ROS can promote cell proliferation and differentiation, whereas, excessive amounts of ROS can interfere with cellular signaling pathways by causing oxidative damage to lipids, proteins and DNA^22^,^23^. Furthermore, ROS could affect various signaling pathways such as MAPK signal transduction cascades. c-Jun N-terminal kinase (JNK), a stress-activated protein kinase of the MAPK family, plays vital roles in apoptosis, autophagy and some other cellular events^24^,^25^. Studies have demonstrated that the activation of JNK signaling triggered by mitochondrial ROS was involved in the mechanism of drug ototoxicity^26^,^27^. ROS/JNK signaling pathway has been reported to mediate cell death in cochlea cells and proved to be a promising drug target in the treatment of deafness^28^.

While a series of publications on the role of NLRX1 in different cell types *in vitro* have appeared in literature, the effects of NLRX1 on auditory cells in inner ear, however, have been studied only sparingly. Recently, we preliminarily demonstrated that NLRX1 may be related to aging- and neomycin-induced deafness *in vivo*^29^. However, the precise mechanisms underlying the actions of NLRX1 on auditory cells in response to the ototoxic drugs are still unclear. This promotes us to investigate whether NLRX1 plays roles in cisplatin-elicited ototoxicity. Herein, we propose that under the condition of cisplatin stress, the survival of cochlea cells may be affected by altered NLRX1 via regulating ROS/JNK pathways associated with cell death. To test this hypothesis, the present study was designed to explore the effects of NLRX1 on auditory model HEI-OC1 cells, a conditionally immortalized cochlear cell line derived from the mouse organ of Corti^30^, treated with cisplatin, and to elucidate the underlying mechanisms.

## Results

### NLRX1 is expressed in HEI-OC1 cells

**Figure 1:**
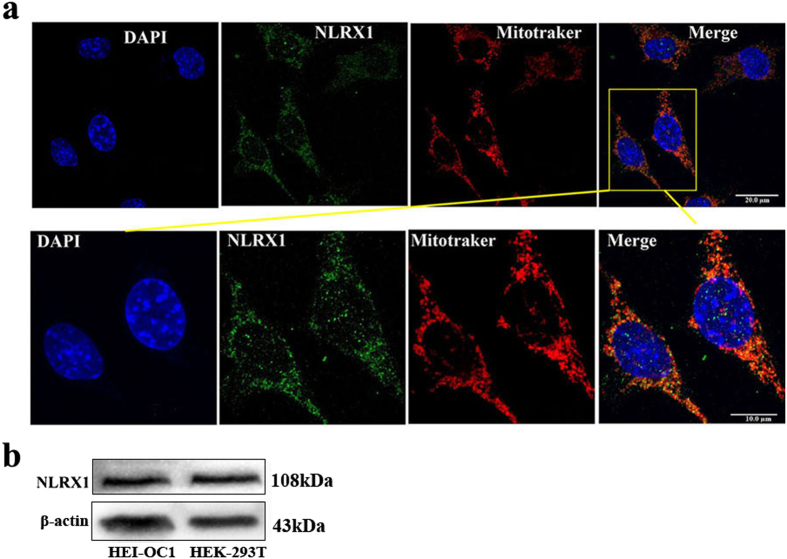
NLRX1 expression in HEI-OC1 cells. (**a**) Representative immunofluorescence staining of NLRX1 (green) in HEI-OC1cells. Localization of NLRX1 protein to mitochondria showing that NlLRX1 co-localized with mitochondria. Confocal images of cells labeled green for NLRX1 and stained red with mitotracker-deep red. Merge showing co-localisation of NLRX1 and mitotracker (orange). All the nuclei were stained with DAPI (blue). (**b**) Western-blot analysis of NLRX1 protein expression in HEI-OC1 and HEK-293T (positive control) cells.

As it was still unknown whether NLRX1 existed in HEI-OC1 cells, therefore, the present work initially investigated the basic status of NLRX1 expression by Immunofluorence. We observed the punctate expression of NLRX1 (green staining) in cytoplasm of the HEI-OC1 cells and NLRX1 was localized to mitochondria (red staining) (Fig. 1a). This observation is in consonance with earlier reports on NLRX1 expression pattern. Western blot also proved the expression of NLRX1 in HEI-OC1 cells (Fig. 1b). Similar NLRX1 expression was observed in HEK-293 cells, which was served as the positive control of NLRX1 expression. These findings confirm the existence of NLRX1 in HEI-OC1 cells, thereby providing an essential foundation for our subsequent study of NLRX1 in auditory cells *in vitro*.

### Cisplatin reduces cell viability via inducing apoptosis in HEI-OC1 cells

**Figure 2:**
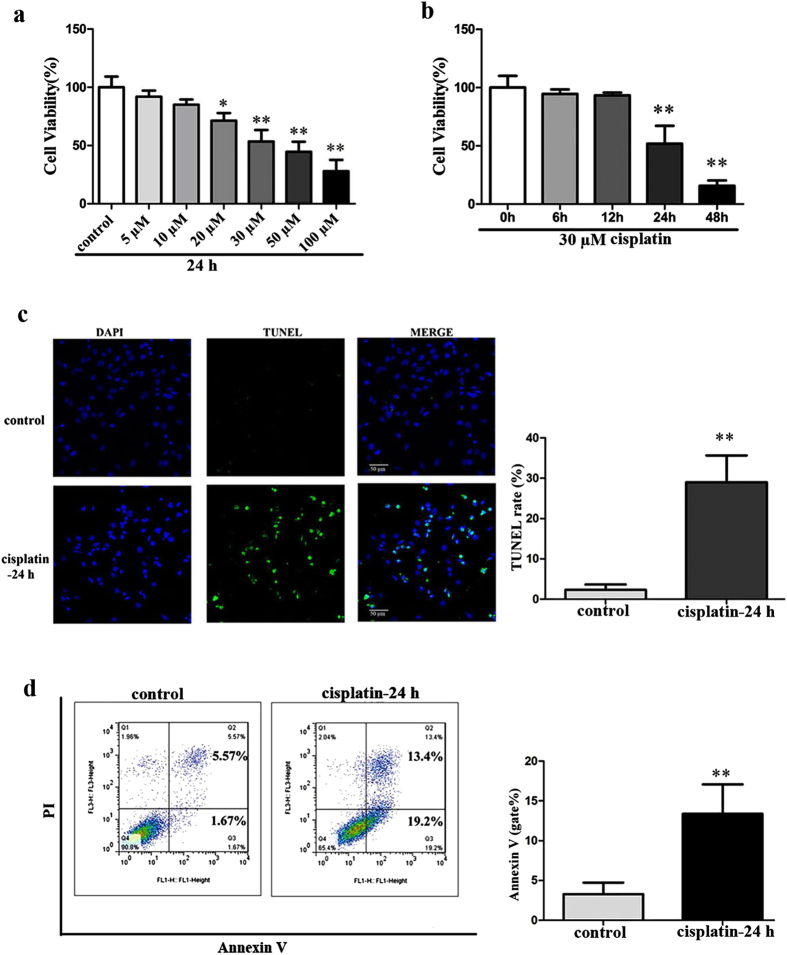
Cisplatin induced cytotoxity via apoptosis in HEI-OC1 cells. (**a**) Cells were treated with the designated concentration of cisplatin for 24 h, n = 6. (**b**) Cells were treated with 30 μM cisplatin for the designated periods of time, n = 6. MTT assay showed that the decrease in cell viability with cisplatin treatment appeared in a concentration- and time-dependent manner. The concentration of 30 μM cisplatin was selected for the following experiments. Data were indicated as mean ± SEM (*P < *0.05* and **P < *0.01* versus cisplatin untreated groups, determined using one–way ANOVA). (**c**) Cells treated with 30 μM cisplatin for 24 h were evaluated by TUNEL assay. Apoptotic cell nuclei were stained TUNEL positively (green), and all of the nuclei were stained with DAPI (blue). The number of TUNEL-positive cells increased significantly after cisplatin treatment, n = 4. Data were indicated as mean ± SEM (**P < *0.01* versus control, determined using an independent t-test). (**d**) Flow cytometry plots of AnnexinV/PI staining cells, Annexin V negative and PI negative quadrant (lower left quadrants, Q4) defines viable cells, Annexin V positive and PI negative quadrant (lower right quadrant, Q3) defines early apoptotic cells, Annexin V positive and PI positive quadrant (upper right quadrant, Q2) defines late apoptotic cells and necrotic cells. The proportion of apoptotic cells (gate %) in the lower right quadrant (Q3) increased significantly after cisplatin treatment, n = 3. Data were indicated as mean ± SEM (**P < *0.01* versus control, determined using an independent t-test).

As evidenced by MTT assay, cisplatin-treatment decreased cell viability of HEI-OC1 cells in a concentration- and time-dependent manner (Fig. 2a,b). Cell viability was markedly reduced approximately to 53.4 + 4.955% after 24 h-treatment of 30 μM cisplatin, and this treatment pattern was selected for the following experiments. Cell apoptosis was assayed by TUNEL, and the number of TUNEL-positive HEI-OC1 cells was visibly increased in cisplatin-treated group than that in control group (Fig. 2c). Moreover, similar changes were obtained by flow cytometry of double Annexin V/PI staining cells, in which the proportion of apoptotic cells was significantly higher in cisplatin-treated cells than that in control group (Fig. 2d). These data indicate that cisplatin exerts its ototoxicity mainly through induction of apoptosis in HEI-OC1 cells.

### Cisplatin increases NLRX1 expression and ROS generation in HEI-OC1 cells

**Figure 3:**
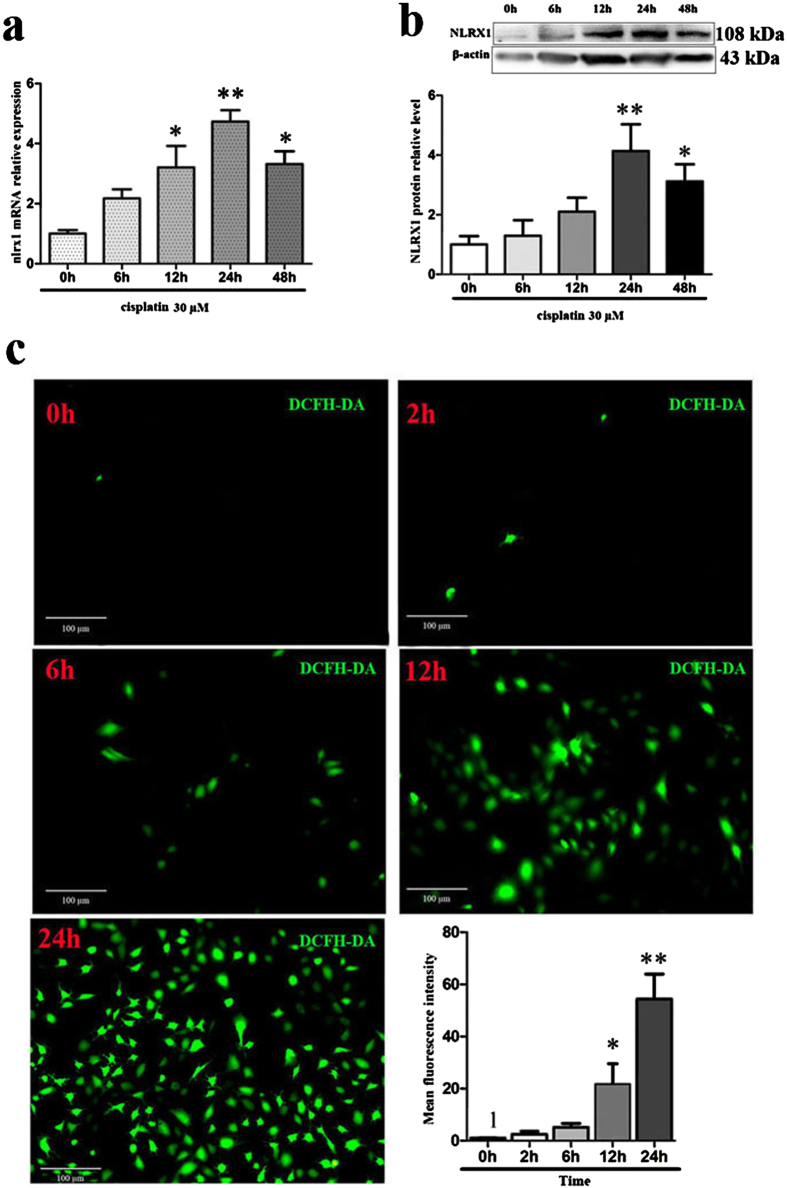
NLRX1 expression and ROS generation were increased by cisplatin in HEI-OC1 cells. After treatment with 30 μM cisplatin for the designated periods of time, the mRNA and protein expressions of NLRX1 were both increased in a time-dependent manner in HEI-OC1 cells. (**a**) NLRX1 mRNA expressions were evaluated by qRT-PCR, n = 5. (**b**) Western-blot analysis also represented the increased NLRX1 protein expression with cisplatin treatment, n = 3. All the data were indicated as mean ± SEM (*P < *0.05* and **P < *0.01* versus 0 h, determined using one–way ANOVA). (**c**) The level of intracellular ROS was monitored using a peroxide-sensitive fluorescent probe, DCFH-DA staining, fluorescent signal was taken with fluorescence microscope and analyzed with Image J software. ROS was increased by cisplatin in a time-dependent manner, n = 3. All the data were indicated as mean ± SEM (*P < *0.05* and **P < *0.01* versus 0 h, determined using one–way ANOVA).

As previous studies showed that NLRX1 functioned as a regulator of cell death in response to different physiological or pathological stress^15^,^19^, the expression of NLRX1 was examined in HEI-OC1 cells in response to the stimulation of 30 μM cisplatin at different time points of 0 h, 6 h, 12 h, 24 h, or 48 h, respectively. Results of real-time PCR assay showed that, NLRX1 mRNA expression in cisplatin-treated group was obviously enhanced at 18 h, 24 h, 48 h time points and peaked at 24 h (Fig. 3a). Similar expression pattern of NLRX1 protein was observed by Western blot (Fig. 3b). Since NLRX1 is believed to modulate the generation of ROS and the latter was known to potentiate cisplatin-induced ototoxity^16^,^31^, the intracellular ROS level was monitored by DCFH-DA staining at different time points (0 h, 2 h, 6 h, 12 h, 24 h) in HEI-OC1 cells exposed to cisplatin. As shown in Fig. 3c, ROS generation was enhanced in a time-dependent manner after cisplatin treatment and peaked at 24 h, which coincided with the peak time of NLRX1 expression and was opposite with the variation trend of cell viability. That is, cisplatin affects NLRX1 expression and ROS generation in HEI-OC1 cells, and the latter two peak at the same time point, i.e., 24 h, which is also the median lethal time point of cell viability assay. These findings led to the hypothesis that NLRX1 might affect survival of HEI-OC1 cells exposed to cisplatin through regulating intracellular ROS generation.

### NLRX1 potentiates cisplatin-induced apoptosis in HEI-OC1 cells

**Figure 4:**
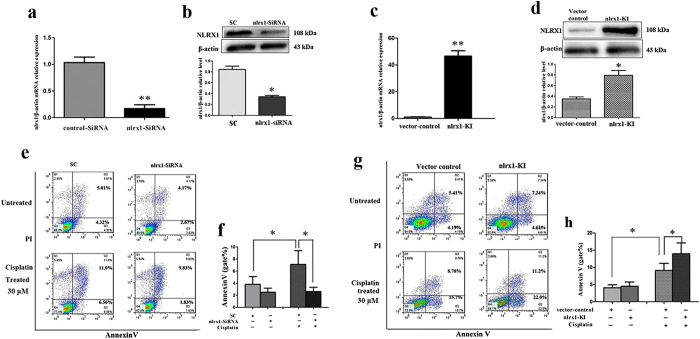
Effects of NLRX1 on Cisplatin-induced apoptosis in HEI-OC1 cells. (**a**) NLRX1 mRNA expressions in nlrx1-siRNA and SC cells after transfection for 24 h were evaluated by qRT-PCR, n = 4. (**b**) NLRX1 protein expressions in nlrx1-siRNA and SC cells after transfection for 48 h were evaluated by western-blotting, n = 3. (**c**) NLRX1 mRNA expressions in nlrx1-KI and vector-control cells after transfection for 24 h were evaluated by qRT-PCR, n = 4. (**d**) NLRX1 protein expressions in nlrx1-KI and vector-control cells after transfection for 48 h were evaluated by western-blotting, n = 3. All data were indicated as mean ± SEM (*P < *0.05* and **P < *0.01* versus sc or vector control group, respectively, determined using an independent t-test). (**e**) The nlrx1-siRNA and SC cells were treated with or without 30 μM cisplatin for 24 h, flow cytometry plots of AnnexinV/PI staining cells. (**f**) The proportion of apoptotic cells (lower right quandrant, gate%) was significantly decreased in nlrx1-siRNA cells after cisplain exposure compared with that of the SC group, n = 4. Data were indicated as mean ± SEM (*p < *0.05*, determined using an independent t-test). (**g**) The nlrx1-KI and vector-control cells were treated with or without 30 μM cisplatin for 24 h, flow cytometry plots of AnnexinV/PI staining cells. (**h**) The proportion of apoptotic cells was significantly increased in nlrx1-KI cells after cisplain exposure compared with that of the vector control group, n = 3. Data were indicated as mean ± SEM (*P < *0.05*, determined using an independent t-test).

To study the relationship between NLRX1 and cell death in response to cisplatin stimulus in HEI-OC1 cells, NLRX1-silenced (nlrx1-siRNA) and NLRX1-knock-in (nlrx1-KI) HEI-OC1 cells were generated. The cells transfected with scrambled siRNA (SC) or vector only (vector-control) served as controls. Results showed that the expressions of NLRX1 mRNA and protein were successfully silenced or overexpressed in the according constructed cells (Fig. 4a,b,c,d). To detect the effect of cisplatin on apoptosis in cells with different NLRX1 expressing conditions, flow cytometry was performed and the proportion of apoptotic cells in the lower right quadrant of dot graph was evaluated. As shown in Fig. 4e–h, both the deficiency and overexpression of NLRX1 exerted no significant effect on cell apoptosis in untreated cells (resting condition), whereas, NLRX1 deficiency decreased the apoptotic proportion in cisplatin-treated cells (stress condition) (Fig. 4e,f, *p < 0.05) and overexpression presented an opposite role (Fig. 4g,h, *p < 0.05), suggesting that NLRX1 deficiency may induce cells under stress condition to be more resistant to apoptosis and cell damage (Supplemental Fig. 1) and its overexpression might sensitize cells under stress condition to apoptosis. These results suggest that NLRX1 could promote apoptosis in cells under stress state but does not appear to have direct effective function on cells under resting condition.

### NLRX1 activates mitochondrial apoptotic pathway in HEI-OC1 cells after cisplatin treatment

**Figure 5:**
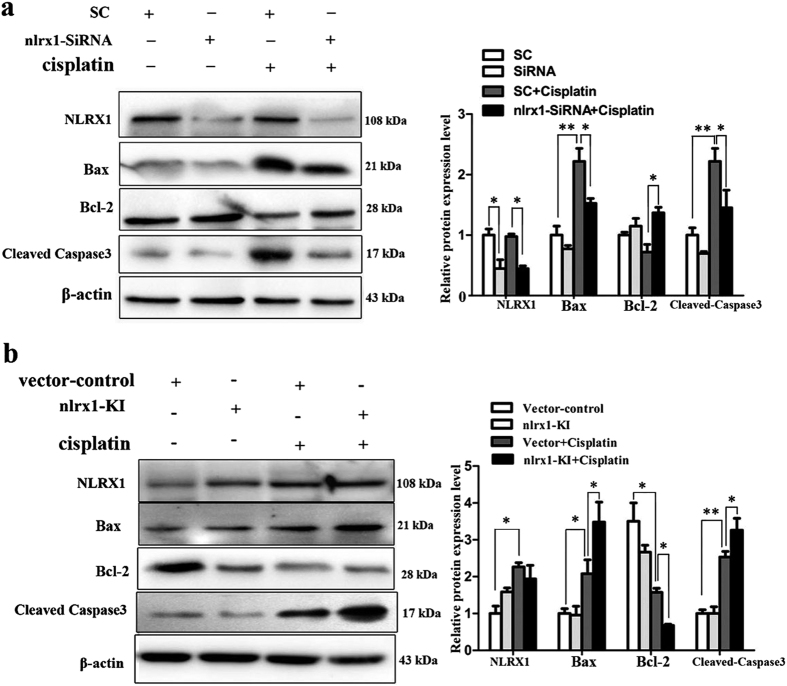
NLRX1 mediated mitochondrial apoptosis in HEI-OC1 cells after cisplatin treatment. (**a**) The nlrx1-siRNA and SC cells were treated with or without cisplatin for 24 h, Western-blot analysis was performed using antibodies against Bax, Bcl-2, Cleaved Caspase3, and β-actin served as controls, n = 3. (**b**) The nlrx1-KI and vector-control cells were treated with or without cisplatin for 24 h, Western-blot analysis was performed using the same antibodies as in (**a**), n = 3. On the right side of each panel, densitometric analyses of the corresponding blots were shown. Data were indicated as mean ± SEM (*P < *0.05* and **P < *0.01*, determined using an independent t-test).

Now that the above results suggest that cisplatin exerts its ototoxicity mainly through induction of apoptosis in HEI-OC1 cells and NLRX1 promotes apoptosis in cisplatin-treated cells, the molecular mechanism by which NLRX1 makes the cells sensitive to apoptosis after cisplatin treatment is explored. Bax, caspase-3 and Bcl-2 are reported to be mainly involved in the mitochondrial apoptotic pathway. As shown in Fig. 5a, Bax and cleaved caspase-3 (an activated form of caspases-3) expressions were significantly decreased in nlrx1-siRNA cells following cisplaitn treatment with an obvious increase of Bcl-2. Instead, NLRX1 overexpression resulted in opposite changes in these three proteins in nlrx1-KI cells (Fig. 5b). Taken together, these data show that mitochondrial apoptotic pathway is activated in cells exposed to cisplatin and NLRX1 could promote the activation in response to cisplatin insult.

### NLRX1-mediated sensitization of cisplatin-induced apoptosis is associated with ROS/JNK activation

**Figure 6:**
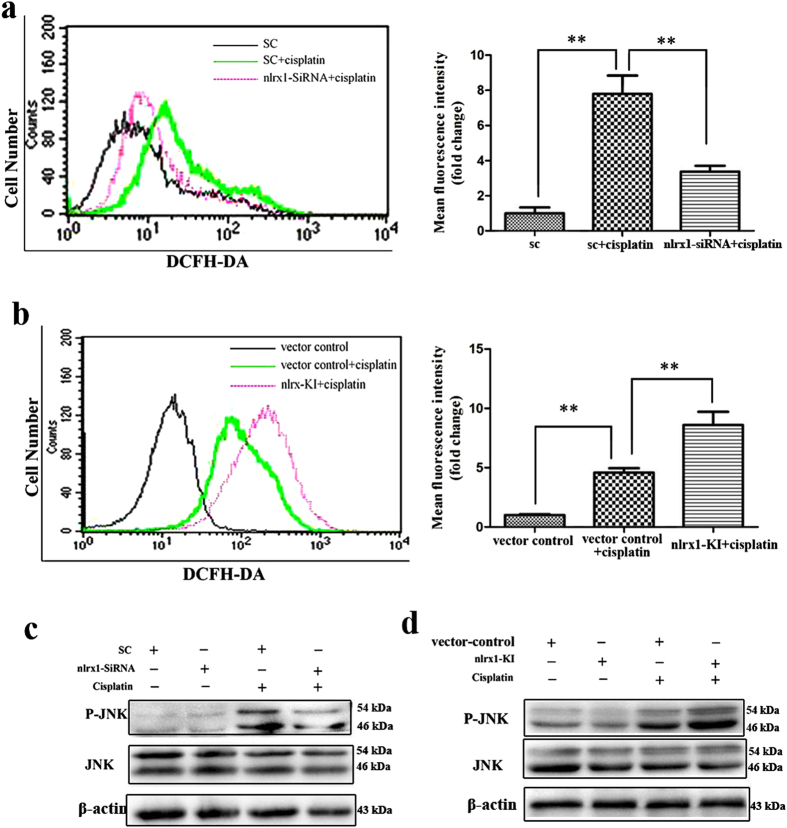
NLRX1 potentiated ROS production and JNK signal activation with cisplatin treatment. (**a**) nlrx1-siRNA and SC cells were labeled with DFCH-DA probe after cisplatin exposure for 24 h and the intracellular ROS levels were evaluated by flow cytometry as described in Materials and Methods. On the right side, the bar graph represented the relative mean fluorescence intensity, n = 3. Data were indicated as mean ± SEM, (**P < *0.01*, determined using an independent t-test). (**b**) The nlrx1-KI and vector-control cells were treated with cisplatin for 24 h and the ROS levels were calculated as above, n = 3. (**c**) The nlrx1-siRNA and SC cells were treated with or without cisplatin for 24 h, Western-blot analysis was performed using antibodies against P-JNK, JNK, and β-actin served as controls, n = 3. (**d**) The nlrx1-KI and vector-control cells were treated with or without cisplatin for 24 h, Western-blot analysis was performed using antibodies against p-JNK, JNK, and β-actin, β-actin served as controls, n = 3.

**Figure 7:**
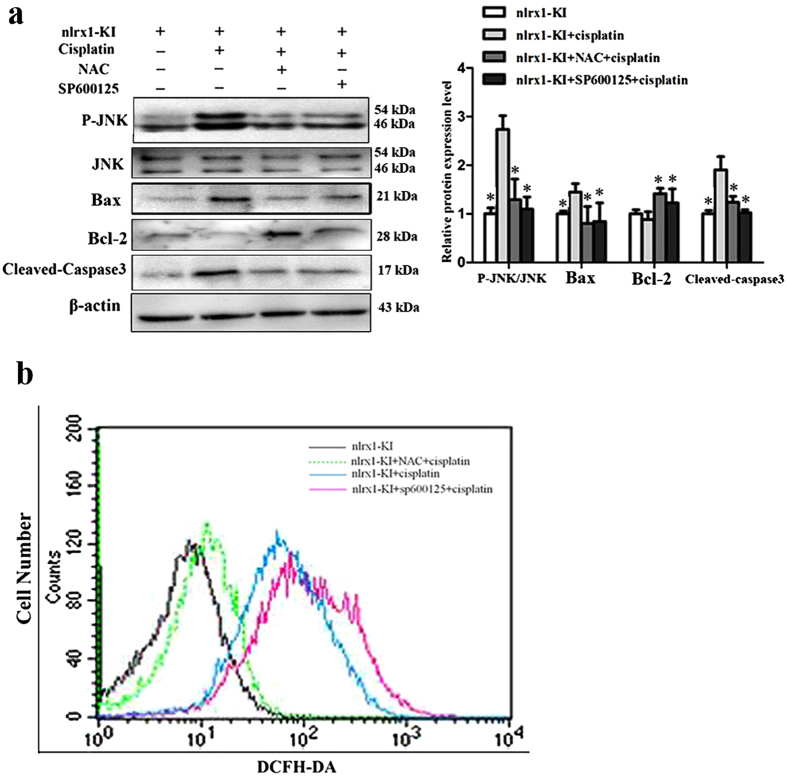
NLRX mediated ROS production and JNK signal activation is associated with sensitization of cisplatin-induced apoptosis. Nlrx1-KI cells were pre-incubated with 5 mM NAC or 10 μM SP600125 for 1.5 h, followed by incubation with 30 μM cisplatin for 24 h. (**a**) Western-blot analysis was performed using antibodies against Bax, Bcl-2, cleaved Caspase3, and β-actin served as controls. On the right side, densitometric analysis of the corresponding blot is shown, n = 3 (*p < *0.05*, by an independent t-test compared with cells treated with cisplatin only). (**b**) Intracellular ROS levels of the four different groups of HEI-OC1 cells were also analyzed by flow cytometry as described in Materials and Methods.

Since NLRX1 was reported to amplify JNK pathway by inducing ROS production under stress state and ROS/JNK signaling was associated with cisplatin-induced ototoxicity^3^,^17^,^32^, the NLRX1-ROS-JNK relationship was determined in cisplatin-treated HEI-OC1 cells. As expected, ROS production was significantly decreased in nlrx1-siRNA cells after cisplatin treatment (Fig. 6a), whereas, increased in nlrx1-KI cells (Fig. 6b), respectively. This indicates that NLRX1-upregulated ROS production is triggered by cisplatin. Next, the effect of NLRX1 on JNK pathway was determined. The p-JNK expression level was significantly reduced in nlrx1-siRNA cells (Fig. 6c), whereas, increased in nlrx1-KI cells (Fig. 6d) after cisplatin treatment, respectively, implying that NLRX1-potentiated JNK activation was triggered by cisplatin. Furthermore, to confirm the role of ROS and JNK activation in NLRX1-mediated sensitization of cisplatin-induced apoptosis, NLRX1-KI cells were exposed to cisplatin either alone or in combination with an intrinsic ROS scavenger (NAC) and specific JNK inhibitor (SP600125) for 24 h. As illustrated in Fig. 7a, both NAC and sp600125 pretreatment significantly abrogated the activation of caspase-3 and Bax expression but increased the level of Bcl-2. These data indicate that NLRX1-mediated sensitization of cisplatin-induced apoptosis is dependent on ROS/JNK pathway which potentiates mitochondrial apoptosis in HEI-OC1 cells exposed to cisplatin. Interestingly, we also observed that NAC effectively suppressed the NLRX1-potentiated phosphorylation of JNK, however, the NLRX1-upregulated ROS generation was not blocked by SP600125 pretreatment as that was done by NAC treatment (Fig. 7b). These findings suggest that ROS appear in the upstream of JNK pathway.

### Up-regulation of NLRX1expression, activation of ROS/JNK and induction of apoptosis in cochlear explants occur in response to cisplatin exposure

**Figure 8:**
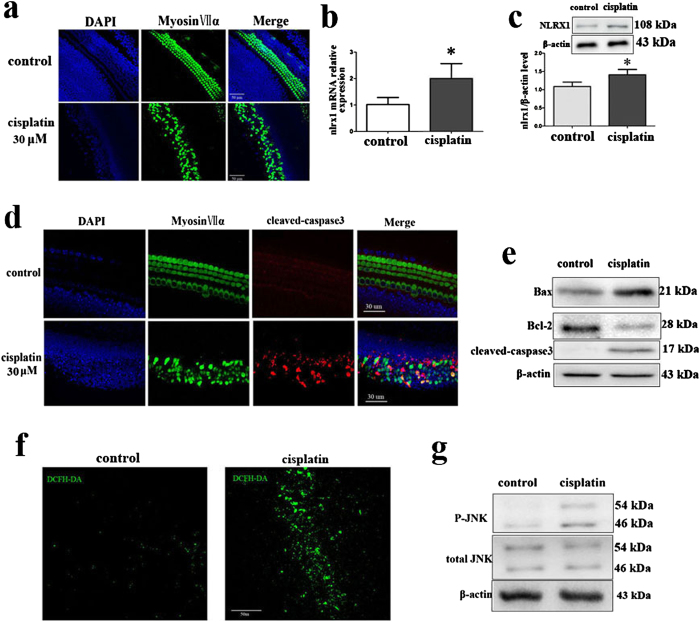
Up-regulation of NLRX1expression, activation of ROS/JNK and induction of apoptosis in Corti explants occurred in response to cisplatin exposure. (**a**) The middle turn of mouse cochlear explant was subjected to immunofluorescence after treatment with 30 μM cisplatin for 24 h. (**b**) RT-PCR results showed that NLRX1 expression was increased in the mouse cochlear explants, n = 3. (**c**) Western blot results showed that NLRX1 expression was increased after cisplatin treatment in the cochlear explants, n = 3. (**d**) Apoptotic cells in cochlear explants were determined by active caspase-3, n = 3. (**e**) Western-blot analysis was performed using antibodies against Bax, Bcl-2, Cleaved Caspase3, and β-actin served as controls, n = 3. (**f**) ROS levels were evaluated by DFCH-DA staining. (**g**) JNK activation was analyzed by western-blot using P-JNK, JNK antibodies and β-actin served as controls, n = 3.

Next, we did certain relevant experiments in cochlear explants of C57BL/6 mouse in order to strengthen the findings from HEI-OC1 cells in *vitro*. To examine the expression of NLRX1 in cochlear explants after cisplatin treatment, the cochlear explants were exposed to 30 μM cisplatin for 24 h. Immunoflourescence staining showed that 30 μM cisplatin treatment for 24 h led to conspicuous loss of hair cells in the middle turn of cochlea (Fig. 8a). qPCR and western-blot analysis showed that cisplatin significantly increased the expression of NLRX1 at both the mRNA and protein levels (Fig. 8b, *p* < 0.01; Fig. 8c, *p* < 0.05). Those were consistent with the results obtained from the HEI-OC1 cells. Now that cisplatin could increase NLRX1 expression, we then detected apoptotic hair cells in cochlear explants (Fig. 8d). As shown in Fig. 8d, the substantial activation of cleaved caspase-3 in cochlear explants treated with cisplatin occurred in comparison to that in the control group. This indicates that cisplatin also exerted its ototoxicity through induction of apoptosis in cochleae. As shown in Fig. 8e by western-blot assay, expressions of Bax and cleaved caspase-3 were significantly increased following cisplaitn treatment with an obvious decrease of Bcl-2, suggesting that mitochondrial apoptotic pathway was activated in cochlear explants exposed to cisplatin. DCFH-DA staining on the cochlear explants showed that generation of ROS was obviously increased after cisplatin treatment for 24 h (Fig. 8f), which was in consonance with the results in HEI-OC-1 cells. Western-blot result also showed that cisplatin activated JNK signal pathway evidenced by high level of P-JNK in cochleae after cisplatin treatment (Fig. 8g).

## Discussion

Any drug, with the potential to cause toxic reactions to structures of the inner ear, is considered ototoxic. Cisplatin is such a typical agent capable of inducing ototoxicity, which not only limits its anti-tumor efficacy, but significantly compromises the life quality of cancer survivors as well. However, the exact mechanism (s) by which cisplatin induces ototoxicity has not been fully elucidated to date. This present study represents the natural extension of our preliminary studies on the interaction of NLRX1 with cisplatin in auditory cells^29^ so as to gain further insight into the intrinsic relationship between NLRX1 and ototoxic drugs.

In this study, we first determined the expression pattern of NLRX1 in auditory model HEI-OC1 cells and found that NLRX1 was localized to mitochondria. This validates the existence of NLRX1 in HEI-OC1 cells, thereby providing the precondition for the following exploration of NLRX1 effects on HEI-OC1 cells *in vitro*. Next, the effects of cisplatin on cell viability and apoptosis were assessed in this work. We demonstrated that cisplatin decreased the cell viability of HEI-OC1 cells in a concentration- and time-dependent manner. Meanwhile, the exposure of HEI-OC1 cells to cisplatin led to appear the typical characteristics of cells undergone apoptosis. These indicate that cisplatin is able to inhibit the growth of HEI-OC1 cells *in vitro* via triggering apoptotic pathway, which is consistent with previous studies in inner ear^33^,^34^.

Previously, studies indicated that intracellular signals might be involved in the development of cisplatin-induced ototoxicity^35^,^36^. Recently, inflammatory damage is thought to contribute to the pathogenesis of cisplatin-induced ototoxicity^37^ and inhibition of pro-inflammatory cytokines significantly attenuates cisplatin-induced damage^38^. Moreover, mitochondrion is emerging as a critical signaling platform for the assembly of signalosomes regulating the apoptotic and inflammatory pathways^39^. In present study, we found that cisplatin increased NLRX1 expression and ROS generation in HEI-OC1 cells. Interestingly, the two indexes, NLRX1 expression and ROS production, shared the same peak time, 24 h, while, changed oppositely as compared with cell viability after cisplatin treatment. It has been reported that NLRX1 can regulate cell death, mitochondrial function and ROS production in different cell types in response to different stimuli^15^,^16^,^18^. In this work, NLRX1 expression was significantly increased along with enhancement of ROS generation in HEI-OC1 cells exposed to cisplatin. Considering the decreased cell viability aforementioned, we found that increase of NLRX1 expression was accompanied by cell degeneration with cisplatin exposure. This indicates that the enhancements of NLRX1 and ROS are negatively correlated with cell viability in cells treated with cisplatin. The above result indicates that cisplatin could trigger the intracellular ROS generation that was associated with ototoxicity, which might be potentiated by enhancement of NLRX1 expression in response to cisplaitn. In this regard, we hypothesized that NLRX1 might promote cisplatin-induced cell death through influencing ROS generation in HEI-OC1 cells.

To study the relationship between NLRX1 and cell death in response to cisplatin stimulus in HEI-OC1 cells, NLRX1-silenced and NLRX1-overexpressed HEI-OC1 cell lines were successfully constructed. Both the deficiency and overexpression of NLRX1 exerted no significant effect on cell apoptosis in resting cells, whereas, NLRX1 deficiency decreased the apoptotic proportion in cisplatin-stimulated cells and overexpression exerted an opposite effect, suggesting that NLRX1 deficiency might induce cells under stress condition to be more resistant to apoptosis, whereas, its overexpression might sensitize cells under stress condition to apoptosis. Recently, certain researchers have identified a role for NLRX1 in the regulation of cell death in various cellular systems through different pathways^15^,^19^. Imbeault *et al*. demonstrated that NLRX1 redirects cellular stress towards apoptosis to protect cells from necrosis-like cell death in neuron cells^15^. Lei *et al*. demonstrated that NLRX1 acts as a positive regulator of autophagy during antiviral signaling^13^. Our results indicate that NLRX1 acts as an important regulator of cisplatin-induced-ototoxity by accelerating apoptotic pathway.

Now that the above results suggest that cisplatin exerted its ototoxity mainly through induction of apoptosis in HEI-OC1 cells and NLRX1 promotes apoptosis in cisplatin-treated cells, the molecular mechanism by which NLRX1 makes the cells sensitive to apoptosis after cisplatin treatment is explored subsequently. One of the two apoptotic pathways, the mitochondrial apoptosis, is reported to be regulated by the combined actions of the pro- and anti-apoptotic members of the Bcl-2 family^40^. Bax, activated caspase-3 and Bcl-2 are assumed to be mainly involved in the mitochondria apoptosis pathway^41^. Our results showed that the expressions of Bax and activated caspase-3 in NLRX1 silencing cells were down-regulated, while, the expression of Bcl-2 was up-regulated significantly in response to cisplaitn treatment and, vice versa, suggesting that NLRX1 sensitized HEI-OC1cells to cisplatin-induced apoptosis dependent on mitochondrial apoptosis pathway.

It has been well established that ROS/JNK signaling pathway has been reported to mediate cell death in cochlear cells and proven to be a promising drug target in the treatment of deafness^28^. JNK, a stress-activated protein kinase of the MAPK family, plays vital roles in apoptosis and some other cellular events^42^,^43^. Since NLRX1 was reported to amplify JNK pathway by inducing ROS production under stress state and ROS accumulation was associated with cisplatin-induced ototoxity^17^, the NLRX1-ROS-JNK relationship was determined in cisplatin-treated HEI-OC1 cells. As expected, we observed that NLRX1 upregulated ROS production and potentiated JNK activation, which is elicited by cisplatin stimulus, suggesting that NLRXL sensitizing HEI-OC1 cells to cispaltin induced death had a crosstalk with ROS/JNK activation. In the current study, we also revealed that enhancement of ROS caused by cisplatin was reduced significantly in NLRX1 silencing cells and, conversely, ROS generation was increased markedly in respond to NLRX1 overexpression followed by cisplatin treatment. This indicates that NLRX1 might influence the mitochondrial function in HEI-OC1 cells, which could further influence the mitochondrial pathway signaling. Recently, a number of studies have demonstrated that the activation of JNK signaling triggered by ROS was involved the mechanism of cisplatin-induced ototoxicity^26^,^27^,^44^. Several studies specifically identified the JNK signaling pathway as a mediator of apoptosis in hair cells. JNK can be activated in response to oxidative stress and able to induce apoptosis in response to cisplatin insult. In this study, in HEI-OC1 cells, NLRX1 could promote JNK phosphorylation after cisplatin exposure, as evidenced by the higher level of P-JNK in NLRX1 overexpression cells and lower level of P-JNK in NLRX1 silencing cells. Based on these findings, we propose that NLRX1 promotes ROS/JNK signal activation, which may subsequently activate the cell damage signal. Indeed, our conclusion was strengthened by result of ROS/JNK signal activation involving in mitochondria apoptosis pathway caused by cisplatin in HEI-OC1 cells.

Lastly, certain relevant experiments in cochlear explants of C57BL/6 mouse were performed in this work. We found that cisplatin treatment resulted in the up-regulation of NLRX1expression, activation of ROS/JNK and induction of apoptosis in cochlear explants. The findings were in consonance with those obtained from HEI-OC1 cells, thereby further strengthening the results from HEI-OC1 cells in *vitro*. It should be pointed out that these experiments were mainly conducted in HEI-OC1 cells, which are frequently used to study drug ototoxicity^45^. In this regard, the precise mechanism underlying the action of NLRX1 in regulation of cisplatin-elicited ototoxity in hair cells needs be studied further.

## Conclusions

In summary, NLRX1 augment cisplatin-induced ROS generation and JNK activation that specifically sensitizes HEI-OC1 cells stimulated by cisplatin, which is mainly associated with mitochondrial apoptotic pathway. These findings provide evidence, for the first time, that NLRX1 acts as an important regulator of the cisplatin ototoxity, which sheds new light on mechanism underpinning the drug-induced ototoxicity.

## Materials and Methods

### Cell culture

HEI-OC1 cells, an auditory cell line derived from the mouse organ of Corti, which possess hair cell-like properties, express several specific molecular markers of hair cells, including Math1 and Myosin VII α^30^. Cells were cultured in high-glucose DMEM (Gibco BRL, Grand Island, NY, USA) supplemented with 10% fetal bovine serum (Gibco BRL, Grand Island, NY, USA) at 33 °C and 5% CO_2_ in a humidified atmosphere without antibiotics.

### Mitotracker staining and Immunofluorescence

HEI-OC1 cells were grown on glass coverslips and incubated with 200 nM Mitotracker-deep Red FM (M22426, Life technologies) at 37 °C from light for 30 minutes, and then were fixed in 4% paraformaldehyde (PFA) for 30 minutes. Cells were permeabilized in PBS with 0.2% triton-X 100 for 10 min at room temperature, blocked with 1% BSA in PBS for 1 h, incubated with primary anti-NLRX1 (17215-1-AP, Proteintech) at 4 °C over night, washed three times in PBS, and incubated with the secondary fluorescent antibody for 1 h in dark at room temperature. Nuclei were stained with DAPI (D9542, Sigma). Specimens were observed under a laser scanning confocal microscope (TSC SPE, LEICA).

### Assessment of cell viability

Cells were seeded at a density of 8 × 10^3^ cells/well in a 96-well plate and cultured over night. Then the cells were treated with cisplatin (Sigma-Aldrich) at the indicated concentrations for 24 h or at the designed concentration for indicated time periods. After washing with PBS gently, cells were incubated with MTT solution at 0.5 mg/ml in dark for 4 h. DMSO was added to solubilize the formazan after removing the supernatant. Absorbance at 570 nm was measured using an ELISA reader (Multiskan MK3) for cell viability and average OD in control cells was taken as 100% of viability. A final concentration of 30 μM cisplatin was selected to damage the HEI-OC-1 cells in the following experiments.


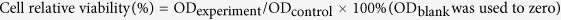


### TUNEL assay

The TUNEL assay was conducted utilizing the Click-iT^®^ Plus TUNEL Assay (Life technologies, USA) according to the manufacturer’s protocol. Briefly, HEI-OC1 cells that were given the indicated treatment were fixed with 4% PFA in PBS for 15 min at room tempreture and then washed with PBS. Then, the cells were permeabilized with 0.1% Triton X-100 in PBS for 10 min, and stained with TUNEL working solution for 1 h at 37 °C in dark. Cell nuclei were stained with DAPI. The specimens were visualized using a Leica confocal laser scanning microscopy.

### Flow cytometry analysis for apoptosis

An Annexin V kit (BD Pharmingen™, RUO) was used for apoptosis analysis. Briefly, cells were collected and washed twice with PBS, and then resuspended in 1× binding buffer at a concentration of 1 × 10^6^ cells/ml. AnnexinV and propidium iodide (PI) were added to cell suspension and incubated at room temperature for 15 minutes in dark. The experimental data (20,000 events per sample) were collected in a FACS Calibur system (BD Biosciences) and then evaluated with the FlowJo 7.6 software.

### siRNA and cDNA Transfection

For siRNA transfection, a target-specific 21-nucleotide NLRX1 siRNA (nlrx1-siRNA) (GenePharma, Shanghai, China) was designed to knock down the gene expression. HEI-OC1 cells were seeded at 0.2 × 10^6^ cells/well in a 6-well plate and allowed to attach overnight. Cells were then transfected in 2 ml Opti-MEM (Gibco BRL, Grand Island, NY, USA) containing 9 μl Lipofectamine 3000^TM^ (Invitrogen, Carlsbad, Ca, USA), with 200 nM siRNA duplexes, following the manufacture’s protocols. The sense strand of NLRX1 siRNA as follow: 5′-GCGACAACCUGAUUCAAAUTT-3′. Transfection control was generated using a scrambled siRNA control (SC). After 24 h of transfection, the medium was removed and replaced with complete growth medium. The transfection efficiency was assessed by quantitative reverse transcription-polymerase chain reaction (qRT-PCR) and Western-blot. Cells were cultivated with cisplatin after transfection for 36 h, and then harvested for western blot and flow cytometry assays.

For NLRX1 overexpression, NLRX1 knock-in cells (nlrx1-KI) were generated using the plasmid DNA (pEX2-nlrx1) (GenePharma, Shanghai, China). HEI-OC1 cells (about 0.3 × 10^6^ cells/well in a 6-well plate) were transfected with 3 μg of the plasmid pEX2-nlrx1 DNA and 9 μL of the Lipofectamine 3000^TM^ in Opti-MEM, following a protocol provided by the manufacturer. Transfection control (vector control) was generated using empty plasmid vectors. The Opti-MEM medium was replaced 24 h later with complete growth medium. Cells were treated with cisplatin for 24 h after transfection for 48 h, and then harvested for western blot and flow cytometry assays. If required, 10 μM SP600125 (a specific JNK inhibitor) (Sigma-Aldrich) or 5 mM NAC (a intrinsic ROS scavenger) (Sigma-Aldrich) was added 1.5 h prior to cisplatin treatment to inhibit the JNK pathway or ROS accumulation, respectively.

### Total protein extraction and Western blot

After the designed treatment, culture medium was discarded and cells were gently washed with cold PBS, then, lysed in RIPA (P0013B, Beyotime Institute of Biotechnology) buffer. The protein concentrations were detected using the BCA protein assay kit (Shenergy Biocolor Bioscience & Technology Company, Shanghai, China). Equal amounts of proteins were loaded on and seperated by 8%~12% SDS-PAGE electrophoresis, then, transferred to polyvinylidene fluoride membranes (PVDF; Immobilon-P, Cat. No. IPVH00010). Membranes were blocked in TBS containing 0.05% tween20 (TBST) with 5% BSA, and incubated with primary antibodies overnight at 4 °C. After washed with TBST, the membranes were incubated with secondary antibodies and the protein signal was detected using a chemiluminescence solution, ECL kit (Millipore, USA). The intensity of protein bands was quantified using Image J software (Broken Symmetry Software, USA). β-actin was used as loading control. The primary antibodies were used as follows: rabbit anti-p-JNK (9251S, Cell Signaling Technology), rabbit anti-total-JNK (9252S, Cell Signaling Technology), mouse anti-Bcl-2 (2876S, Cell Signaling Technology), rabbit anti-cleaved-Caspase3 (9664S, Cell Signaling Technology), Rabbit anti-NLRX1 (17215-1-AP, Proteintech), rabbit anti-BAX (ab32503, Abcam, Cambridge, UK) and mouse anti-β-actin (TA-09, ZSGB-BIO).

### Quantitative RT-PCR

Total RNA was extracted from HEI-OC1 cells and Corti explants with Trizol (Invitrogen, USA) according to the protocol of the manufacturer. The relative expression level of NLRX1 mRNA was measured by reverse transcription and real-time PCR. β-actin was used to normalize the NLRX1 mRNA expressions. 1 μg of total RNA was reverse-transcribed with Revert Aid First Strand cDNA Synthesis Kit (Burlington, Ontario, Canada) as recommendation by the manufacturer. Quantitative PCR was performed in 25 μl reactions containing 12.5 μl 2× SYBR Premix EX Taq (Takara, Dalian, China), 1 μl cDNA template, 1 μl forward primer, 1 μl reverse primer, deionized water complemented the rest volume. The real-time PCR parameters were pre-degeneration at 95 °C for 3 min then 40 cycles of degeneration at 95 °C for 50 s, annealing at 58 °C for 45 s, elongation at 72 °C for 50 s. Primers sequences as the following: NLRX1 forward primer: 5′-CCTCTGCTCTTCAACTTGCTC-3′, NLRX1 reverse primer: 5′-CCCATCTGATCCAGAACA-3′, β-actin forward primer: 5′-GTCCCTCACCCTCCCAAAAG-3′, β-actin reverse primer: 5′-GCTGCCTCAACACCTCAACCC-3′. The levels of mRNA were normalized to β-actin. The expression of genes was analyzed by method of 2^−ΔΔCt^.

### Detection of ROS

Cellular ROS level was measured by DCFH-DA staining (D6883, Sigma Technologies) according to manufacturer’s instructions. Briefly, cells were seeded in 6-well plates at a density of 2.5 × 10^5^ cells/well and treated with the designate conditions. After washing with pre-warmed serum-free DMEM, cells were incubated with 10 μM DCFH-DA in serum-free medium for 30 minutes, followed by washing twice with PBS. Fluorescent signal intensity was taken with fluorescence microscopy or flow cytometry. Flow cytometry analyses (20,000 events per sample) were performed in FACS Calibur system with extinction and emission at 485 and 538 nm and then evaluated with the Cell Quest software.

### Culture of cochlear explants

C57BL/6J mice were killed on postnatal day 3, and the cochleae were carefully dissected out. The striavascularis and spiral ligament were dissected away, leaving the organ of Corti and seeded intact on a glass coverslip coated with Cell-Tak (BD Biosciences, Franklin Lakes, NJ, USA). The cochlear explants were treated with or without 30 μM cisplatin for 24 h, and then, subjected to immunofluorescent staining, qRT-PCR and western-blot analysis. All animal procedures were approved by the Animal Care Committee of Shandong University, Jinan, P.R. China (NO. ECAESDUSM 20123011). All procedures were carried out in accordance with the approved guidelines.

### Statistical analyses

Data were presented as mean ± SEM. One-way ANOVA or t-test was used to analyze statistical significance of the results. *p* < 0.05 was considered statistically significant.

## Additional Information

**How to cite this article**: Yin, H. *et al*. NLRX1 accelerates cisplatin-induced ototoxity in HEI-OC1 cells via promoting generation of ROS and activation of JNK signaling pathway. *Sci. Rep.* **7**, 44311; doi: 10.1038/srep44311 (2017).

**Publisher's note:** Springer Nature remains neutral with regard to jurisdictional claims in published maps and institutional affiliations.

## Supplementary Material

Supplementary Information
