# Supplementary material for: NLRX1 accelerates cisplatin-induced ototoxity in HEI-OC1 cells via promoting generation of ROS and activation of JNK signaling pathway
Source: Sci Rep. 2017 Mar 13;7:44311. doi: 10.1038/srep44311 (PMC5347132; doi:10.1038/srep44311)
Supplement: Supplementary Information [file srep44311-s1.pdf]

Supplementary Information

NLRX1 accelerates cisplatin-induced ototoxicity in HEI-OC1 cells via promoting generation of ROS and activation of JNK signaling pathway

Haiyan Yin[a,b], Gaoying Sun[a,c], Qianqian Yang[a,b], Chen Chen[a,b], Qi Qi[a,b], Haibo Wang[a,c], Jianfeng Li[a,b,c,*]

a Department of Otolaryngology-Head and Neck Surgery, Shandong Provincial Hospital Affiliated to Shandong University, Jinan, 250021, P.R. China

b Department of Pathology and Pathophysiology, Shandong University, Cheeloo Healthy Science Center, Jinan, 250012, P.R. China

c Shandong Provincial Key Laboratory of Otology, Jinan, 250021, P.R. China

***Corresponding author: Jianfeng Li**

Department of Otolaryngology-Head and Neck Surgery, Shandong Provincial

Hospital Affiliated to Shandong University, Shandong Provincial Key Laboratory of

Otology, Jinan, 250021, P.R. China

Tel.: +86-531-85187583

Fax: +86-531-87980304

email: lijianfeng@hotmail.com

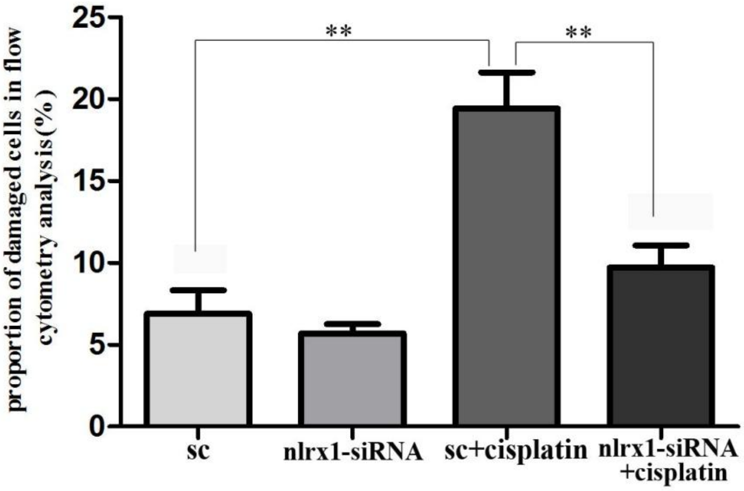

Supplemental Fig. 1. **Downregulation of NLRX1 expression increased the tolerance of HEI-OC-1 cells to cisplatin**

The nlrx1-siRNA and SC cells were treated with or without 30 μM cisplatin for 24 h, flow cytometry result showed that the proportion of damaged cells(consist of early apoptotic cells, later apoptotic cells and necrotic cells)was significantly increased by cisplain. The proportion of damaged cells was significantly decreased in nlrx1-siRNA cells after cisplain exposure compared with that of cisplatin treated SC cells, n=4. Data were indicated as mean ±SEM (***p<0.01*, determined using an independent t-test).
